# Drivers and outcomes of between-group conflict in vervet monkeys

**DOI:** 10.1098/rstb.2021.0145

**Published:** 2022-05-23

**Authors:** Miguel Gareta García, Miguel de Guinea, Redouan Bshary, Erica van de Waal

**Affiliations:** ^1^ Inkawu Vervet Project, Mawana Game Reserve, KwaZulu Natal 3115, South Africa; ^2^ Department of Eco-Ethology, Faculty of Biology, University of Neuchâtel, Rue Emile Argand 11, Neuchâtel 2000, Switzerland; ^3^ Movement Ecology Laboratory, Alexander Silverman Institute of Life Sciences, Department of Ecology, Evolution and Behavior, The Hebrew University of Jerusalem, Jerusalem 91904, Israel; ^4^ Department of Ecology and Evolution, Faculty of Biology and Medicine, University of Lausanne, Lausanne 1015, Switzerland

**Keywords:** between-group conflict, vervet monkeys (*Chlorocebus pygerythrus*), normalized differentiation vegetation index (NDVI), core areas, group size

## Abstract

Neighbouring groups compete over access to resources and territories in between-group encounters, which can escalate into between-group conflicts (BGCs). Both the ecological characteristics of a territory and the rival's fighting ability shape the occurrence and outcome of such contests. What remains poorly understood, however, is how seasonal variability in the ecological value of a territory together with fighting ability related to the likelihood of between-group encounters and the extent to which these escalate into conflicts. To test this, we observed and followed four vervet monkey groups in the wild, and recorded the group structure (i.e. size, composition), the locations and the outcomes of 515 BGCs. We then assessed key ecological measures at these locations, such as vegetation availability (estimated from Copernicus Sentinel 2 satellite images) and the intensity of usage of these locations. We tested to what extent these factors together influenced the occurrence and outcomes of BGCs. We found that the occurrence of BGCs increased at locations with higher vegetation availability relative to the annual vegetation availability within the group's home territory. Also, groups engaging in a BGC at locations far away from their home territory were less likely to win a BGC. Regarding group structure, we found that smaller groups systematically won BGCs against larger groups, which can be explained by potentially higher rates of individual free-riding occurring in larger groups. This study sheds light on how the ecology of encounter locations in combination with a group's social characteristics can critically impact the dynamics of BGCs in a non-human primate species.

This article is part of the theme issue ‘Intergroup conflict across taxa’.

## 1. Introduction

In many social species, groups of individuals defend resources like food, water or sleeping sites against neighbours (see also [1,2]). The benefits of resource defence must be weighed against the costs in the form of time, energy, injury or even death [3–7]. A variety of studies have investigated what factors affect an individual's decision to participate in a between-group conflict (BGC), and how the sum of individual decisions translates into the group winning or losing. Group size emerges as one key factor. To date, most studies reported either a positive effect of group size on the likelihood of winning a contest ([8–12]; see review: [13]) or no obvious effect of group size [12,14].

Despite group size being a key determinant of winning or losing BGCs, other factors may have an influence. Most importantly, groups are more likely to win a conflict if it takes place within or close to their ‘core area’ [15], i.e. the most frequently used parts of their home range [8,10–12,14,16,17]. Core areas presumably represent particularly valuable locations [8], and familiarity and knowledge of an area's value are assumed to increase the group members' willingness to fight [18]. The resulting increased probability of winning is called the ‘Bourgeois effect’ [19]; in contrast, individuals are expected to behave cautiously in unfamiliar or unknown terrain and hence the group becomes less likely to win [11].

Various ecological variables may also affect individual propensity to fight and hence a group's probability of winning a conflict. For example, the motivation of a group to engage in BGCs at a given location can vary across annual cycles of food availability [20]. Food resources are often asymmetrically distributed in time and space across landscapes [21,22]. Winning BGCs at locations with a high density of food resources is, therefore, beneficial [23]. Animal groups may assess the ecological value of a given location based on the seasonal fluctuations of its food productivity but also depending on the overall food availability within their home range. This can affect considerably the individuals' motivation to engage in BGCs at such locations ([20]; but see [24,25]). Alternatively, there are also demographic factors like female reproductive status that may affect the occurrence and outcome of BGCs. In vervet monkeys, for example, having an infant strongly reduces female participation in BGCs [26].

Here we build on previous research to elucidate the environmental and social drivers of BGC in vervet monkeys. Vervet monkeys regularly face between-group encounters that often cause BGC, here defined as acts of aggression between members of different groups. Many BGCs merely consist of threat displays and displacement without physical contact but in some cases, aggression is fierce and can lead to severe injuries. Winning BGCs in this species appears to be beneficial in terms of securing important resources such as food patches or sleeping sites. A conflict typically consists of several waves of attack and counterattacks, interrupted by sessions of within-group social interactions [27]. The most active participants are the philopatric adult females, who use grooming (as a reward for participation) and/or aggression (as a punishment for not participating) to get immigrant adult males to participate as well ([27]; see also discussion by [28]). BGCs are frequent as vervet monkeys do not defend exclusive territories but may fight over specific resources within overlapping home ranges [26,29,30]. Nevertheless, close to 50% of between-group encounters do not lead to BGCs according to our definition as no overt aggression is observed [25].

**Figure 1.**
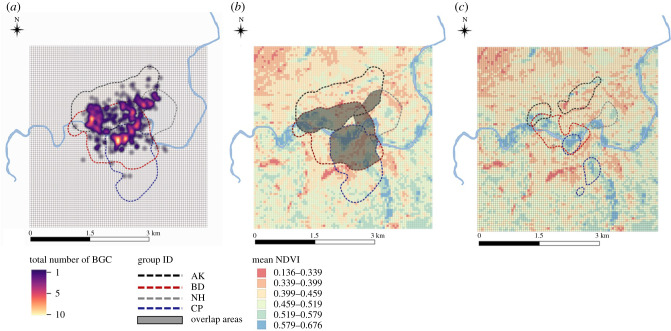
Study area (Inkawu Vervet Project and Hylonyane river, South Africa) divided in to 50 m^2^ quadrats showing data from 2016 until 2019 of: (*a*) between-group conflict (BGC) density at 10 m radius; (*b*) mean Normalized Differentiation Vegetation Index (NDVI) together with the study groups' home ranges (95% kernel density estimates); and, (*c*) mean NDVI together with the study groups’ core areas (50% kernel density estimates).

We used data from 515 BGCs in four groups of wild vervet monkeys, in the Mawana Game Reserve (KwaZulu-Natal, South Africa) to identify environmental and social factors that drive the occurrence of BGCs and impact which group wins the conflict. On a basic level, we aimed to test whether we could reproduce results from other studies showing that distance from the core area and relative group size affect the probability of winning or losing. In order to evaluate the environmental conditions promoting BGCs and affecting their outcome, we estimated the density of vegetation as a proxy for food availability inside the entire study area, using a technique called Normalized Differentiation Vegetation Index (henceforth ‘NDVI’) as a proxy of plant productivity (see Methods) [31–34] and, therefore, a proxy for food availability ([26,33,35]; figure 1). We then evaluated whether BGC locations represented an above-average food value across one or several of three timescales: (i) the absolute maximum value in a study year; (ii) the average value over a study year; and (iii) the relative average value on the day of the BGC. Our working hypotheses were that the relative short-term and/or long-term food value of a location affects the occurrence of BGCs, and which group is more likely to win, possibly in interaction with relative group size (i.e. larger groups are more likely to win when the contested food source is of higher value). As an additional social factor besides group size, we also considered the groups' reproductive status by counting the number of infants (i.e. individuals younger than 1-year old) present in the group at the time of the encounter [36]. As mothers invest significant time and resources into each of their offspring, and infants are particularly vulnerable group members [25,37,38], mothers are expected to be risk-averse during BGCs [25,39], which would reduce their group's overall fighting power. Therefore, we predicted that groups would be less likely to win BGCs when their current number of infants is relatively high.

## 2. Methods

### (a) Study site and study subjects

We collected the data at the Inkawu Vervet Project (IVP), in the Mawana Game Reserve (KwaZulu-Natal, South Africa: 28°00′ S, 031°12′ E) from January 2016 to October 2019, over greater than 2500 h of observation time. Our study subjects were wild vervet monkeys habituated to human presence. For the present study, we selected four neighbouring groups: Ankhase (AK), Baie Dankie (BD), Crossing (CR) and Noha (NH). Group size ranged between 17 and 67 individuals per group including all age classes, of which between 4–21 were adult females (mean ± s.d.; 10.5 ± 2.5 individuals per group; table 1). Researchers were able to identify the study subjects at the individual level using facial and body features, and trained to collect behavioural data with a minimum inter-observer reliability agreement of 80% Cohen's Kappa [40].

**Table 1.** Basic information on the four vervet monkey study groups (AK, BD, CR and NH). (Mean group size and s.d., home range size (kernel density estimation (KDE) 95) and core area size (KDE 50) in hectares. Percentages indicate core area size in relation to each groups' home range size.)

group	group size (mean ± s.d.)	KDE 95 (home range size)	KDE 50 (core area)	
AK	28.81 ± 7.40	215.59	65.10	(30.20%)
BD	55.49 ± 7.31	265.49	60.85	(22.92%)
CR	37.17 ± 5.51	184.35	40.83	(22.15%)
NH	37.76 ± 6.26	101.64	24.52	(24.13%)

### (b) Data collection on space use

One female per group was fitted with a VHF (very high frequency) collar allowing us to rapidly find each group. Observations took place from 06.00 until 18.00 in summer and from 08.00 until 17.00 in winter, which corresponded to the time period where monkeys were active during the day [41]. We collected GPS data as part of our behavioural observations on the study groups, using handheld devices (Palm Zire 22 and HP Travel Companion iPAQ rx5935) with Pendragon 5.1 software. The total number of GPS points was 3584 (AK = 875; BD = 1167; NH = 1383; CR = 159).

Based on the GPS data, we estimated the study groups' home ranges using the R package adehabitatHR 0.4.15 and the kernel density estimation (hereafter ‘KDE’) method [42]. We defined a group's home range as the 95% KDE isopleth (figure 1*b*) and core area as the 50% KDE isopleth (figure 1*c*; [33]). For the smoothing factors, we chose the smallest integer that resulted in a continuous isopleth without holes (*h* = 90–125; as in [43]), which provided better estimates than those from using the reference or least-squared cross validation smoothing factors.

In order to match space use data with estimates of the ecological value of different locations within the study area (see next section), we generated a map consisting of 50 m^2^ quadrats covering the home ranges of the four study groups (*n* quadrats per group: AK = 1047; BD = 1210; CR = 849; NH = 487). For each group, we calculated the intensity of use of a given quadrat by dividing the number of GPS locations we collected within it by the total number of GPS locations we collected across the home range of the study group (i.e. akin to the ‘marginality index’: [44,45]).

### (c) Estimating the food value of grid cells

**Figure 2.**
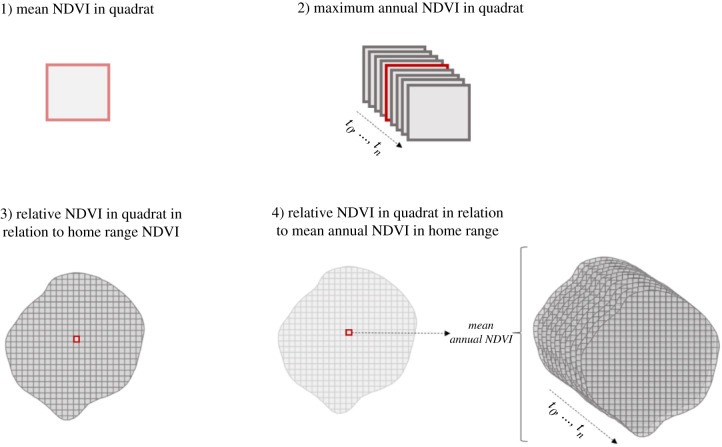
For each quadrat in the study area, we calculated first the mean NDVI per recorded date in order to then determine the maximum annual NDVI, the current NDVI of a contested quadrat relative to the mean current NDVI of the entire home range during on the day of a BGC (or from the satellite image closest in time), and the relative annual NDVI of contested quadrats relative to the mean annual NDVI of the entire home range.

To estimate the food value of each quadrat within the study area, we used images captured by the Copernicus Sentinel 2 satellite, which was launched by the European Space Agency in 2015. Sentinel 2 takes snapshots of the Earth surface at 14-day intervals with a resolution of 10 m^2^ [46]. To enhance the accuracy of our vegetation availability estimation, we opted for satellite images with a cloud cover below 15% [32]. We used these images to calculate the mean NDVI (figure 2), i.e. the mean of five 10 m^2^ pixel values that yield the 50 m^2^ units for each quadrat and each recorded date. NDVI is an index that can range from −1 to +1 [47], based on measures of the coefficient between earth surface reflectance patterns in the red and near-infrared regions of the electromagnetic spectrum. Plants absorb the visible (red) light for photosynthesis but reflect the near-infrared light. Thus, the more reflected radiation there is in near-infrared wavelengths compared to visible wavelengths, the higher the vegetation density estimate is for a given 10 m^2^ pixel on the satellite image. In turn, vegetation density is assumed to be correlated with food sources for vervet monkeys (i.e. fruits, flowers, leaves and invertebrates).

We calculated three metrics derived from the mean NDVI measurements to estimate the food value of quadrats in which encounters took place relative to all quadrats of a group's home range: (i) absolute maximum NDVI per year (hereafter ‘maximum annual NDVI’) as the maximum NDVI recorded in each quadrat for each study year; (ii) relative NDVI per year (hereafter ‘relative annual NDVI’) as the mean annual NDVI recorded in a given quadrat divided by the mean annual NDVI of the group's entire home range; and, (iii) current relative NDVI on an encounter day (hereafter ‘relative current NDVI’) as the mean NDVI recorded in a given quadrat during an encounter day divided by the mean NDVI of the group's home range during the same day (figure 2). Given that the satellite only takes snapshots every two weeks, we used the snapshot closest in time to the encounter day as the best possible approximation. On average, the timeframe between a group encounter and its associated satellite image was 8.97 ± s.d. 7.45 days. To summarize, we estimated the maximum food value, the average long-term food value and the current food value of each disputed quadrat within the study area. We calculated all metrics using QGIS 3.12.2 (see the electronic supplementary material, table 1S with defined concepts).

### (d) Between-group conflicts

Whenever a followed group (the ‘focal group’) was within 100 m of another group and showing signs of perceiving its presence, we recorded a between-group encounter [26,48]. All other data collection stopped at this point so that the focus was on the encounter. As a first step, we recorded the GPS location, time, and identities of the two groups. We considered that an encounter escalated into a BGC if two or more individuals from the focal group engaged (actively or as targets) in aggressive interactions with members of the other group [49]. Aggression could be very mild, i.e. threat displays (lifting eyebrows), increase in intensity by chasing and in the extreme involve physical contact. Typical conflicts often consisted of waves of chases and counter-chases to interrupt periods of mutual staring/threat displays and within-group grooming [27]. A BGC ended when one of the two groups left the area [13,26]. We considered the group that remained at the BGC's location to be the *winner* while the other group was the *loser* [26]. We considered the conflict a draw if the two groups tolerated each other or if they left in different directions simultaneously [27].

For each BGC, we extracted the current number of adult females as well as the number of infants in both groups from the project's general demography files. We used the number of adult females rather than total group size as we consider this the most ecologically relevant proxy for a group's total fighting ability given that previous research in vervet monkeys has shown that adult females are the most active participants in BGCs [27]. Note that these figures were highly correlated with the number of adult males, total number of adults in the group, and overall group size (see the electronic supplementary material, figure S1). Thus, the interpretation of our results is robust no matter which correlate of total potential fighting ability is used. The number of infants present in the group provided an indicator of the females' reproductive status (mean ± s.d infants per group, AK: 3.83 ± 2.29; BD: 7.45 ± 4.85; CR: 3.59 ± 3.41; NH: 6.33 ± 2.12).

An important preparation for statistical analyses involved the classification of the disputed quadrat according to its usage by the two contesting groups. For the ‘where do BGCs take place’ analyses, we classified for each group whether the quadrat was part of its core area (top 50%), part of its extended home range (51–95%), or outside its home range (final 96–100%, i.e. rare excursions into neighbouring areas). Note that some quadrats were part of the core areas of two different groups (figure 1*c*). For the ‘who wins’ analyses, we followed analyses by previous studies [8,16] and hence calculated the Euclidean distance between each BGC quadrat and the groups' core area borders.

### (e) Statistical analyses

For the statistical analyses, we fitted a set of generalized linear mixed-effects models (GLMMs) with binomial error distributions and logit link functions using the function ‘*glmer*’ from the *lme4* package 1.1–21 in R (R software v. 3.6.1) [50,51]. First, we tested which factors may affect the probability of BGC occurrence in a given quadrat (‘where’ models) with three GLMMs [52]. In all three models, we used the presence/absence of encounters in quadrats as a binary response variable. Our three NDVI estimates (maximum NDVI for model ‘where 1’; relative annual NDVI for model ‘where 2’; and relative current NDVI for model ‘where 3’) were the main predictor variables of interest, analysed separately in the three models. To compare the characteristics of quadrats with group encounters versus quadrats without group encounters (controls), we randomly selected 50 control quadrats across the focal groups' home range for every date in which a BGC occurred. This random sample helped to minimize the potential issues derived from spatial autocorrelation in our dataset (i.e. adjacent quadrats can lead to convergence issues and Type I errors: [53]). In addition to the NDVI factors, we included a fixed set of predictor variables in all three models: (i) core area, i.e. whether or not the quadrat was inside the core area of at least one group; (ii) overlapping area, i.e. whether the quadrat was part of the extended home range areas of both groups (if not one group would have made an ‘excursion’ into the extended home range of the other group); and (iii) number of infants (i.e. individuals younger than 1 year old) present in the group during each BGC. We fitted group identity and date as random factors in this set of models, and we fitted random slopes for all the predictor variables [54,55]. We also included pairwise interaction terms between the different predictors. Given the repeated hypothesis testing across the three models, we conducted a Bonferroni correction that led to an adjusted *p*-value ≤ 0.017 as the new statistical significance threshold.

To evaluate which factors may predict the outcome of BGCs (‘outcome’ model), we ran another GLMM in which winning/losing was the binary response variable. Because relative (proportional) values would be difficult to interpret, we introduced our predictor variables separately for the focal group (i.e. the one that was followed by the observer) and for the encountered group. To give one example, group A would be scored as being half as far away than group B from their respective core areas regardless of whether the actual values were 10 m versus 20 m or 100 m versus 200 m. We fitted the following predictors in this ‘outcome’ model: (i) number of adult females present in each group during each BGC; (ii) number of infants present in each group during each BGC (i.e. hereafter ‘reproductive status’); (iii) distance between encounter location and core area for each group; (iv) relative current NDVI for each group; (v) relative annual NDVI for each group; and (vi) intensity of use of quadrat for each group where an BGC took place. We added group identity as a random factor.

For all four models, we *z*-transformed continuous predictors to a mean of 0 and standard deviation of 1. We visually checked for model assumptions, such as the normal distribution of the models' residuals and variance homogeneity (i.e. qq-plots were plotted against fitted values). To rule out multi-collinearity among variables, we examined the variance inflation factor (vif) of all fitted models by using the function ‘vif’ from the *car* package [56]. Low values indicate low to no multi-collinearity effect (all the vif values in our models were below 1.3; [56]). We also compared each full model to its null version (the same model with only random variables) using likelihood ratio tests (i.e. ‘anova’ function set to Chisq). Additionally, we opted for model selection. When an interaction term had no significant effect, we ran a reduced model by excluding this interaction term and including only the main effects. Finally, if the likelihood ratio test for full and null model comparison was significant, we inspected the significance of each predictor variable using likelihood ratio tests comparing full models with reduced models without that variable, using the ‘drop1’ function [54].

## 3. Results

We recorded a total of 515 BGCs between four different group combinations (i.e. AK-BD = 208; AK- NH = 89; BD-CR = 154; and BD-NH = 64). On average, the home ranges of the study groups covered 206.07 ± 73.08 ha (core area: 46.61 ± 16.95 ha), with important overlaps between groups (table 1 and figure 1*b,c*). A total of 285 BGCs took place within the core area of at least one of the groups (heatmap figure 1*a*). The mean annual NDVI value for the entire area encompassed by the study groups' home ranges was 0.36 ± 0.12, and 0.40 ± 0.14 for the core areas. The mean NDVI value in the quadrats where BGCs took place was 0.42 ± 0.15 (figure 1*b,c*).

### (a) ‘Where’ models: factors predicting the occurrence of between-group conflicts

**Figure 3.**
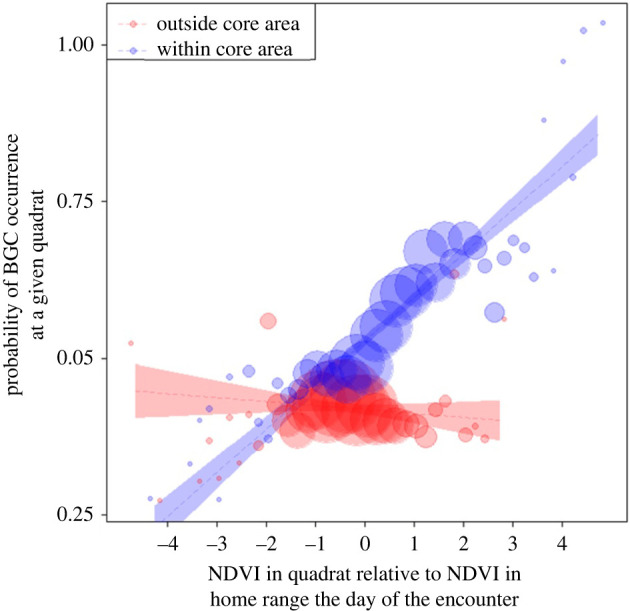
Differences in the probability of BGC in a given quadrat as subject to the mean NDVI in quadrat relative to the mean NDVI in home range during the encounter day. The dashed lines represent the fitted model (with all other predictors being centred), dots represent the averaged probability of having an encounter, and their area corresponds to the number of encounters in the respective quadrat (*n* = 1 to 25 per bin). Shaded areas represent 95% confidence intervals of the fitted models.

The maximum annual NDVI value of a given quadrat did not significantly affect BGC occurrence in that quadrat (likelihood-ratio test model ‘*where 1’*: *χ*^2^_7_ = 8.65, *p* = 0.071, *α′* = 0.017). By contrast, we found that the probability of BGC occurrence in a given quadrat increased when the relative annual NDVI value of the quadrat was higher than the relative annual NDVI value of other quadrats within the focal group's home range (likelihood-ratio test model ‘*where 2’*: *χ*^2^_7_ = 18.56, *p* = 0.009, *α′* = 0.017). Although we did not find evidence that relative daily NDVI value influenced the occurrence of BGC (likelihood-ratio test model ‘*where 3’*: *χ*^2^_7_ = 15.44, *p* = 0.031, *α′* = 0.017; figure 3), we found a trend suggesting that BGC occurrence increased in quadrats with high relative daily NDVI value but only when such quadrats were located inside of the focal group's core area (estimate = 0.376 ± 0.114, d.f. = 1, *p* = 0.026, *α*′ = 0.017; figure 3).

Furthermore, the reproductive status of the group members (i.e. number of infants) did not affect BGC occurrence. Also, the location of the quadrats within home ranges was not significant in any of the three models (i.e. within or outside core areas and within or outside overlapping areas; see the electronic supplementary material, table S2 for details).

### (b) ‘Outcome’ model: factors predicting the probability of winning a between-group conflict

Of the 515 BGCs, 314 had a clear winner/loser outcome (i.e. AK-BD = 138, AK-NH = 48, BD-CR = 89, BD-NH = 41) and could hence be used for further analyses. From model comparison analyses, the model containing all our predictor variables was statistically significant compared to the null model (likelihood-ratio test model *outcome*: *χ*^2^_15_ = 37.69, *p* = 0.001; table 2). The two main results are the following. First, it appears that groups with more females (and hence overall larger groups) are less likely to win an encounter than groups with fewer females. This conclusion derives from the result that the probability of the focal group winning a BGC increased with an increasing number of adult females present in the encountered group during BGC (estimate = −1.36 ± 0.39, d.f. = 1, *p* < 0.001; table 2), while the mirroring analysis (the effect of number of females in the focal group) did not produce a significant result. Overall, the negative effect of female numbers is clear (see the electronic supplementary material, figure S2). As a second major result, the likelihood of the focal group winning a BGC decreased when BGCs took place further away from the core area of the focal group (estimate = 0.32 ± 0.15, d.f. = 1, *p* = 0.028; table 2). Similar to the results regarding the number of adult females, we did not find a significant effect in the mirroring analysis, i.e. the effect of the distance of the encountered group to their core area. The other main factors, i.e. intensity of use of the disputed quadrat as well as current and mean annual relative NDVI of the disputed quadrat did not significantly affect the probability of winning (table 2).

**Table 2.** Summary of the model examining the factors that may affect the probability of winning a BGC. (The two groups have different estimates for the different variables (relative current and mean annual NDVI, distance to core, and intensity of use and core area). The analysis takes the perspective of the focal group (followed by observer). Accordingly, if an estimate is positive, it shows a lower likelihood of the focal group winning a BGC, while a negative estimate indicates a higher likelihood of the focal group winning a BGC. Statistically significant results are shown in italics.)

model outcome (winning/losing): *χ*^2^_15_ = 37.69, *p*-value = 0.001			
predictor	estimate ± s.e.	^CI^lower ^– CI^upper	*p*–value
(intercept)	−0.486 ± 0.152	−0.743 to 0.304	^a^
number of adult females in focal group	−0.060 ± 0.394	−0.8322 to 0.711	0.879
*number of adult females in encounter group*	*−1.361 ± 0.387*	*−2.119 to −0.602*	*<0.001*
relative current NDVI for focal group	0.107 ± 0.255	−0.393 to 0.606	0.674
relative current NDVI for encounter group	−0.015 ± 0.238	−0.481 to 0.450	0.948
relative annual NDVI for focal group	−0.202 ± 0.861	−1.889 to 1.485	0.816
relative annual NDVI for encounter group	0.176 ± 0.857	−1.504 to 1.857	0.839
*distance to core area for focal group*	*0.317 ± 0.146*	*0.031 to 0.603*	*0.028*
distance to core area for encounter group	0.205 ± 0.149	−0.086 to 0.497	0.163
intensity of use by focal group	−0.210 ± 0.171	−0.544 to 0.125	0.217
intensity of use by encounter group	−0.151 ± 0.166	−0.476 to 0.175	0.408
*n* adult females (focal group * encounter group)	−0.220 ± 0.267	−0.631 to 0.191	0.288
relative current NDVI (focal group * encounter group)	*−0.281 ± 0.143*	*−0.561 to −0.001*	*0.029*
relative annual NDVI (focal group * encounter group)	0.124 ± 0.169	−0.207 to 0.456	0.461
distance to core area (focal group * encounter group)	−0.081 ± 0.155	−0.385 to 0.223	0.601
intensity of use (focal group * encounter group)	0.078 ± 0.188	−0.289 to 0.445	0.677

^a^Not shown owing to having a very limited interpretation.

**Figure 4.**
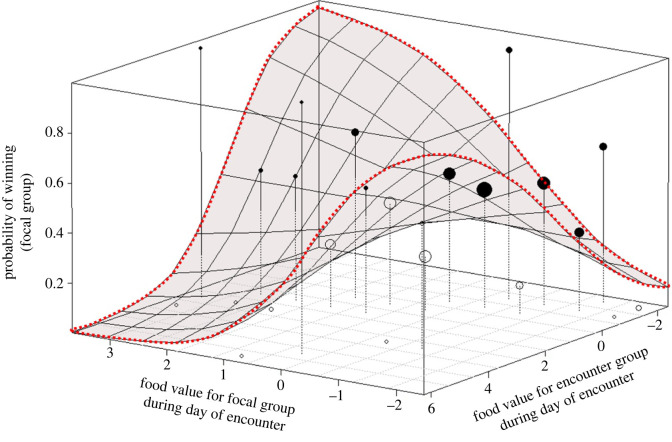
Probability that vervet monkeys win a BGC in relation to the relative food value of the location where the BGC took place during the day of the encounter for the focal group and for the encounter group. The height of spheres represents the probability that the focal group of vervet monkeys would win per combination of the relative daily food value for the focal group and the relative daily food value for the encounter group. Each surface (i.e. square) represents the expected probability to win a BGC according to the model (conditional on all other predictors being set at their average value). Sphere size corresponds to the relative number of observations, with closed circles being above the model surface and open circles below. Note that the model predictions for extreme food value combinations (high food value for one group and at the same time low value for the other group) are extrapolations rather than based on real data.

Finally, we found a significant interaction effect regarding the relative current NDVI value of the disputed quadrat for the focal group relative to the relative current NDVI value of the disputed quadrat for the encountered group (estimate = −0.28 ± 0.14, d.f. = 1, *p* = 0.009; table 2). The model predictions are plotted as a landscape of the focal group's winning probability in figure 4. Note that the values in the corners of the parameter space reflect model extrapolations rather than measured data. The figure shows one result that can be expected and is also reasonably well supported by data, namely that the focal group is most likely to win if the current value of the quadrat is high for the focal group and low for the encountered group (far corner of the graph). The second result is counterintuitive: the focal group is also more likely to win if the relative current NDVI of the contested quadrat is low (negative) for the focal group and high (positive) for the encountered group (front corner of the graph).

## 4. Discussion

We aimed to identify factors that drive the occurrence of BGCs in wild vervet monkeys and/or affect which group wins. The only factor predicting where BGCs are likely to happen was average annual plant density: BGCs happened mostly in quadrats with high annual mean NDVI relative to other parts of the groups' home ranges. Regarding factors that predict the outcome of BGCs, we found that being close to one's own core area increased the probability of winning, while larger groups with a larger number of adult females were more likely to lose. Furthermore, we found a rather complex interaction between the current value of a quadrat relative to the rest of the home range for the focal group versus the current value for the encountered group. Below we discuss the main implications of these results.

### (a) Normalized Differentiation Vegetation Index measures and the occurrence of between-group conflicts

Vervet monkeys do not defend exclusive territories but may fight over specific resources within overlapping home ranges [26]. Our approach of using plant productivity as estimated from satellite images (NDVI) as a correlate of food availability [33] revealed the long-term perspective of resource defence decisions in vervet monkeys: groups were most likely to fight over locations of high average annual value, rather than considering the current or maximal value. This main result is intriguing as it is cognitively much easier to assess the current value of a contested resource through olfaction and visual cues, allowing for rapid decision-making based on immediate benefits [57,58]. Indeed, short-term considerations have been reported in other studies [59,60]. According to our results, vervet monkeys may consider the current value of a location only if it is within the group's core area (strong observed effect albeit statistically a tendency, figure 3). Defending locations that are on average valuable is cognitively more demanding. Information regarding the long-term ecological value of different geographical locations across their home range must be acquired and integrated by vervet monkeys during routine interactions with their environment. A few studies have shown that primates and whales may have detailed memories of important food locations within their territories/home ranges [61–63]. By memorizing relevant foraging events, animals have been argued to construct ‘information gradients' of statistical botanical knowledge that contribute to making efficient decisions regarding where to forage [64,–66]. Our results add to this topic by showing that such past experience may also affect decisions on resource defence. The major importance of the value of specific locations for the occurrence of BGCs in vervet monkeys is also emphasized by the absence of any effect of broader location categories: vervet groups apparently do not defend only their core area or fight over overlapping areas, and the presence of vulnerable group members (infants) does not modulate decisions regarding which locations to defend.

As a note of caution, it is currently unclear to what extent NDVI measures correlate with food availability across different time scales. Potentially, high plant productivity might best capture mean local annual food availability rather than current or peak annual availability. Regarding the latter, supra-annual cycles of tree productivity may shape vegetation oscillations that may not be picked up in our analyses [21,67,68]. Future research can engage with a more detailed assessment by incorporating complementary information, such as measures of soil composition [69,70], underground water flow [71] and long-term ecological predictability of food resources [72].

### (b) Factors affecting between-group conflict outcome

In line with previous evidence reported in the literature, groups engaging in BGCs at locations geographically far away from their core areas were more likely to lose the conflict [10–12,14,16,17]. Core areas are presumably valuable because frequent usage yields more detailed knowledge [8], which in turn leads to the ‘Bourgeois effect’ [19] that the local group is more likely to win.

A surprising result was that smaller groups with fewer adult females were more likely to win BGCs. To date, most studies either reported a positive effect of group size on winning a contest ([8–12]; see review: [13]) or no obvious effect of group size [12,14].

A study on Verreaux's sifakas (*Propithecus verreauxi*) may indicate a way to reconcile our result with the existing literature: in the sifakas, groups of intermediate size were most likely to win BGCs [24]. Lewis and colleagues [24] argued that large groups of sifakas might be unable to solve the public goods problem inherent in BGCs, namely that individuals gain the highest benefit if their group wins without their own contribution (free-riding). At least in cross-species comparisons, larger groups face the problem of individuals free-riding more than smaller groups do, unless the larger or stronger sex is philopatric ([73]; see also [1]). In vervet monkeys the smaller sex, females, are philopatric, and the sizes of our study groups were larger than the group sizes of vervet monkeys at other sites (see the electronic supplementary material, table S2; [74–77]). A possible interpretation of our results is hence that the public goods problem was so prevalent in our comparatively large study groups that they could not mount a proper group attack or defence because of a lack of volunteers and a surplus of free-riders.

A proper assessment of BGCs in our study population, with the use of game-theoretic concepts, could yield more detailed insights that could potentially explain the negative effect of the number of adult females on winning. As it stands, models of public good games typically assume that the acquired benefits are shared equally by all group members ([78–81]; but see [82] for asymmetric sharing), and do not consider any coordination cost as a function of group size. We predict that behavioural observations would reveal that both assumptions are violated in our population. Firstly, we doubt whether contested locations contain enough food to feed 30–50 vervet monkeys. As a consequence, only some group members—typically the highest-ranking females and their offspring—would benefit from winning a contest. Lower ranking adult females would hence have no incentive to participate in BGCs. Secondly, we anticipate higher coordination costs in larger groups. This is because the larger the group, the more likely it should become that members spread out over larger areas in order to find food. The entire large group aggregating to fight for a contested location would hence require more movement costs by individual members compared to a smaller group with less group spread.

Our predictions regarding the sharing patterns of the gained resource, participation as a function of rank, and group spread as a function of group size could all be quantified in future studies. If they were confirmed for our study area but also found to be vindicated in locations or species where group sizes are (much) smaller, our surprising result would fit predictions from established concepts (see also conceptual contributions to N-player games by [1,28]). Alternatively, it is important to point out that our analyses are based only on four group combinations. Therefore, it is conceivable that idiosyncrasies in the largest (BD) and the smallest (AK) groups may have affected participation in BGCs. For example, the strength of social relationships may vary between our study groups, and as a consequence, the release of oxytocin (a neurohormone pivotal to between-group aggression, see [83]).

Apart from the distance to core area and the number of adult females, we found a third significant predictor of winning/losing: an interaction between current NDVI values as indicators of current food availability for the focal (observed) group versus the opponent (the encountered group). The full model prediction of the interaction as illustrated in figure 4 should be interpreted cautiously. Indeed, there is no theoretical explanation as to why the focal group should be most likely to win either if the current value of the contested location is high for the focal group and low for the opposing group *or* if low for the focal group and high for the opposing group. The former makes sense (the value asymmetry favours the focal group) while the latter does not (the value asymmetry should favour the opposing group). The problem with the interpretation of the interaction is that there are hardly any data for the extreme asymmetry in values. This lack of data makes intuitive sense: any NDVI value for a contested quadrat is likely to be either relatively high, intermediate or low for *both* groups. If one group lives in a home range of on-average high plant productivity, the NDVI value of a contested location relative to the rest of the home range will *invariably* be lower than the relative NDVI value for the opposing group that lives in a home range of on-average low plant productivity. Thus, while the analysis yielded a significant result and asymmetries in the value of a contested resource should indeed affect winning probabilities [84], a larger dataset is needed to draw stronger conclusions regarding the importance of resource value asymmetries to BGC outcomes in vervet monkeys. This conclusion is further strengthened by the fact that only current NDVI affected the outcome of contests, while only mean annual NDVI affected the likelihood of BGCs in the first place, which creates a mismatch regarding the role of immediate versus long-term benefits of locations that is difficult to explain.

## Data Availability

All data used in this manuscript are available from the Dryad Digital Repository: https://doi.org/10.5061/dryad.6q573n617 [86].
